# Influence of Relative Humidity on the Mechanical Properties of Palm Leaf Manuscripts: Short-Term Effects and Long-Term Aging

**DOI:** 10.3390/molecules29235644

**Published:** 2024-11-28

**Authors:** Wenjie Zhang, Shan Wang, Hong Guo

**Affiliations:** 1 Key Laboratory of Archaeomaterials and Conservation, Institute of Cultural Heritage and History of Science & Technology, University of Science and Technology Beijing, Ministry of Education, Beijing 100083, China; d202310769@xs.ustb.edu.cn; 2 Chinese Academy of Cultural Heritage, Beijing 100029, China; cnicpbj@gmail.com

**Keywords:** palm leaf manuscripts, mechanical properties, dynamic vapor sorption, thermomechanical analysis, simulated aging experiment, preventive conservation

## Abstract

Palm leaf manuscripts are a valuable part of world cultural heritage. Studying the mechanical properties of palm leaf manuscripts and their changes due to environmental influences is of great significance for understanding the material characteristics, aging mechanisms, and preventive conservation of these manuscripts. This study used dynamic vapor sorption (DVS) and a thermomechanical analyzer (TMA) to investigate the changes to the mechanical properties of palm leaf manuscripts in response to different relative humidity conditions and different time periods. The short-term study results show that exposure to varying relative humidities leads to changes in the equilibrium moisture content (EMC) of palm leaf manuscripts, causing the bending strength of the samples to decrease significantly with increasing humidity. The bending modulus initially increases and then decreases as the humidity increases. Moreover, the greater the desorption hysteresis of the samples, the more pronounced the changes to the mechanical properties. Therefore, a stable environment in terms of humidity can prevent changes in the mechanical properties of palm leaf manuscripts, thereby preventing the onset of degradation. The results of the long-term aging studies indicate that prolonged exposure to either very dry or very humid conditions greatly affects the mechanical properties of palm leaf manuscripts, which is detrimental to their preservation. The samples kept at 50% RH did not exhibit significant signs of deterioration, with no notable changes in their mechanical properties or chemical structure. This suggests that 50% RH is a relatively optimal humidity condition for the preservation of palm leaf manuscripts.

## 1. Introduction

Before the widespread use of paper, palm leaf manuscripts served as a popular literary medium in South Asia and Southeast Asia, carrying rich cultural significance. These manuscripts not only recorded knowledge across various fields, such as history, literature, philosophy, art, and science, but also held an important place in Buddhist culture and religious teachings [1,2,3]. Given their immense historical and cultural value, palm leaf manuscripts are recognized as a precious part of world cultural heritage, making their preservation and preventive conservation especially important.

At present, significant progress has been made in regard to the collection and preservation of palm leaf manuscripts, as well as in research related to cataloging and text interpretation [4,5,6]. Some scholars have conducted systematic analyses of the materials [7,8,9,10] and craftsmanship [11,12] used in the production of palm leaf manuscripts, completed preliminary assessments of degradation [13], and classified common types of damage [14,15]. Based on these studies, conservation professionals have implemented a series of measures [16,17], making important contributions to the preservation and restoration of palm leaf manuscripts. These studies provide foundational data for the scientific understanding and preventive conservation of palm leaf manuscripts and offer valuable references for selecting appropriate preservation environments.

Although there have been some advancements in the research on production techniques, material analysis, and preservation and restoration methods for palm leaf manuscripts, studies on the effects and mechanisms of environmental factors on their aging remain insufficient. Several studies have preliminarily explored the impact of environmental conditions on the physical properties of palm leaf manuscripts. For example, dry environments may reduce the mechanical strength of palm leaf manuscripts [12,18], while excessively high humidity significantly enhances their hygroscopicity [19]. Additionally, in conservation research on other cellulose-based materials like paper and wood, extensive studies have investigated the aging effects and mechanisms of various environmental factors. For instance, high temperatures accelerate the thermal oxidative degradation of cellulose and hemicellulose, resulting in a decline in their mechanical properties [20,21]. Similarly, exposure to high-energy ultraviolet radiation breaks the molecular chains of cellulose, leading to mechanical deterioration and material disintegration [22]. This radiation also induces photochemical reactions in components such as lignin, causing discoloration of the material [23]. Furthermore, acidic gases, like sulfur compounds and nitrogen oxides, react with moisture in the material, triggering acidic hydrolysis [24]. The accumulation of dust directly alters the color and surface properties of the material [25]. These findings offer valuable insights into the preservation of palm leaf manuscripts, particularly for preventive conservation. Notably, as both a literary medium and a cultural heritage artifact, palm leaf manuscripts are typically stored indoors, in libraries or study rooms, thereby largely shielding them from external temperature fluctuations, light exposure, and atmospheric pollutants. Consequently, humidity, which tends to fluctuate more readily, becomes the primary environmental factor to consider in the preservation of palm leaf manuscripts.

Humidity is closely linked to the mechanical properties of palm leaf manuscripts, which directly impact their preservation and restoration processes. Variations in humidity can cause physical deformation of the manuscripts, leading to permanent structural damage [26], while higher humidity levels promote the growth and proliferation of microorganisms [27]. These factors can affect the mechanical properties of palm leaf manuscripts [12] and accelerate material deterioration. Moreover, whether short-term and long-term exposure to the same relative humidity has different effects on the mechanical properties of the manuscripts remains a key question that could lead to the adoption of different approaches to preservation and restoration. Therefore, studying the mechanical properties of palm leaf manuscripts and how they change under the influence of relative humidity is essential for a deeper understanding of their intrinsic characteristics and aging mechanisms. This research is crucial for developing effective restoration techniques and preventive conservation measures.

Dynamic vapor sorption (DVS) can be used to study the hygroscopic behavior of materials in response to different relative humidity conditions. It has shown great potential in regard to evaluating the hygroscopicity of palm leaf manuscripts [28] and other cellulose-based artifacts, such as wood and paper [29,30,31]. Unlike traditional mechanical testing equipment, such as universal testing machines, which require larger sample sizes, a thermomechanical analyzer (TMA) offers advantages in regard to testing mechanical properties using small sample sizes, with high precision and good repeatability [32,33], making it particularly suitable for fragile and precious organic artifacts, like palm leaf manuscripts. However, a TMA lacks intrinsic humidity control capabilities, and traditional humidity-controlled equipment, such as desiccators and constant temperature–humidity chambers, cannot be directly integrated with a TMA. This setup creates a lag between humidity conditioning and mechanical testing, making it challenging to capture the immediate effects of humidity on the material’s mechanical properties. Furthermore, fluctuations in humidity make it difficult to accurately measure the subtle short-term mechanical responses of the material. To overcome these limitations and enable synchronized humidity control and mechanical testing, ensuring real-time accuracy of the mechanical data in response to varying humidity conditions, this study innovatively employs a custom-made Modular Humidity Generator (MHG) connected to the TMA. This method allows precise humidity adjustment and captures the mechanical response of palm leaf manuscripts in diverse humidity environments, providing a scientific basis for studying humidity’s impact on the material’s mechanical properties.

Using raw palm leaf manuscripts as research samples, this study examined the short-term effects of varying humidity on their mechanical properties, as well as the long-term aging effects. This approach aims to deepen our understanding of how environmental factors influence the lifespan of palm leaf manuscripts and provide data that support research on their degradation and preventive conservation.

## 2. Results and Discussion

### 2.1. Hygroscopicity of the Samples

**Figure 1:**
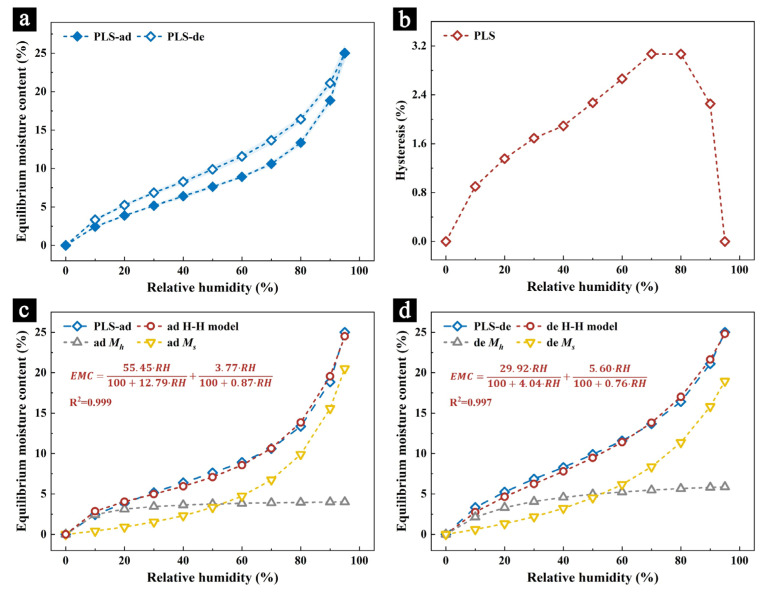
The isothermal adsorption curve of the samples (**a**); the hysteresis curve (**b**); the Hailwood–Horrobin (H–H) model fitting results for the adsorption curve (**c**); and the desorption curve (**d**) of the samples (at 25 °C, 0% RH to 95% RH).

The relationship between the equilibrium moisture content (EMC) of the samples (PLSs) and the environmental relative humidity (RH) is described by an isothermal adsorption curve [34]. The isothermal adsorption curve of the samples is shown in Figure 1a. The curve follows the trend of an IUPAC Type II adsorption isotherm [35]. During the adsorption phase (ad), the EMC of the samples increases with the increasing relative humidity, showing a gradual rise followed by a sharp increase, which is characteristic of the hygroscopic behavior of cellulose-based materials. At 95% RH, the EMC of the samples reached 25.01%. During the desorption phase (de), the EMC decreases as the relative humidity drops, but remains higher than in the adsorption phase, indicating a certain level of desorption hysteresis.

Desorption hysteresis refers to the lag phenomenon that occurs when the behavior of a substance during adsorption and desorption is not identical. This hysteresis is expressed by the hysteresis value, which represents the difference in the equilibrium moisture content (EMC) between the desorption and adsorption phases. The hysteresis curve of the sample is shown in Figure 1b. The hysteresis value of the samples initially increases and then decreases with increasing relative humidity, reaching a maximum value of 3.08% at 70% RH.

To further study the adsorption behavior of the samples, the isothermal adsorption curves were fitted to the Hailwood–Horrobin (H–H) model. Table 1 lists the calculated parameters for each model. The high coefficient of determination (R^2^ > 0.99) confirms the validity of the model.

Using the calculated model parameters, the adsorption curve (Figure 1c) and desorption curve (Figure 1d) of the samples were plotted. The results indicate that the H–H model effectively explains the adsorption and desorption processes of the samples. According to the H–H model, the total adsorbed moisture content in a material can be divided into monolayer moisture content ($M_{h}$) and multilayer moisture content ($M_{s}$) [36]. Typically, in low humidity environments (0% to 40% RH), the adsorption of moisture by cellulose materials is primarily driven by monolayer adsorption. As the relative humidity increases and the monolayer adsorption sites are occupied, monolayer adsorption gradually stabilizes and multilayer adsorption plays an increasingly important role in the moisture adsorption process. This pattern can be observed in both the adsorption and desorption phases of the samples.

During desorption, the $M_{h}$ value of the sample increases compared to the adsorption phase, while the $M_{s}$ value remains unchanged. This suggests that the observed hysteresis may be due to changes in the level of monolayer adsorption between the two processes.

### 2.2. Short-Term Effects of Relative Humidity on the Mechanical Properties of the Samples

#### 2.2.1. Adsorption Process

**Figure 2:**
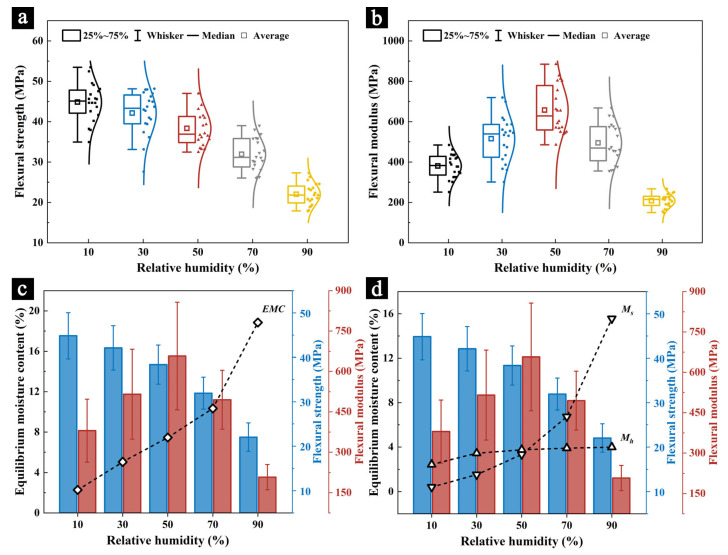
The flexural strength (**a**) and flexural modulus (**b**) of the samples at different humidity levels during the adsorption process, along with the correlations between the flexural strength and modulus with the EMC (**c**), and $M_{h}$ and $\;M_{s}$ (**d**).

The TMA-MHG system was used to simulate and measure the changes in the mechanical properties of the samples as the humidity increased, represented by the flexural strength (Figure 2a) and flexural modulus (Figure 2b). The results indicate that the TMA is a viable method for measuring the mechanical properties of precious and relatively fragile organic materials, such as palm leaf manuscripts. The flexural strength of the samples decreased significantly with increasing humidity, with the highest flexural strength observed at 10% RH (44.86 MPa), while at 90% RH, the flexural strength dropped to only 22.03 MPa. The flexural modulus exhibited a different trend. The highest flexural modulus (657.26 MPa) was observed at 50% RH, while both low and high humidity conditions resulted in a reduced flexural modulus: 380.38 MPa at 10% RH and 208.06 MPa at 90% RH.

This variation aligns with the characteristics of cellulose-based materials. At a low moisture content, the hydrogen bonding between cellulose molecules is stronger, leading to tightly bound fiber bundles and higher overall mechanical strength. However, dry conditions can also increase the brittleness of the material, reducing its flexibility and, thus, its flexural strength or stiffness [37]. In humid environments, cellulose materials absorb large amounts of water and the absorbed water molecules infiltrate the cellulose structure, forming hydrogen bonds with some cellulose molecules. This weakens the intermolecular forces between cellulose chains, significantly reducing the material’s overall mechanical strength. As the moisture content increases, the material becomes more flexible and stiffness decreases, as reflected by the simultaneous reduction in both the flexural strength and flexural modulus [38,39]. Similar phenomena have been observed in regard to other cellulose-based materials, such as wood and paper [37,38,39]. 

The isothermal moisture adsorption curve of the samples (Figure 2c) and the variation of $M_{h}$ and $M_{s}$ with humidity (Figure 2d) further confirm the changes in the mechanical properties of the samples. At a relative humidity of 10% RH, the EMC of the sample was 2.43%, corresponding to the highest flexural strength and a lower flexural modulus. As the humidity increased, the EMC of the sample rose significantly, while the flexural strength gradually decreased and the flexural modulus first increased and then decreased. At 90% RH, the EMC reached 18.85%, at which point both the flexural strength and modulus were at their lowest values.

In low humidity environments (0–40% RH), the EMC of the sample was primarily composed of monolayer moisture content ($M_{h}$), resulting in less pronounced changes in the flexural strength. As the humidity increased, $M_{h}$ stabilized, while the multilayer moisture content ($M_{s}$) increased significantly. The $M_{s}$ value typically reflects the material’s ability to adsorb moisture through physical adsorption and capillary action [40]. This indicates that the physical adsorption and capillary action of the sample increased markedly with increasing humidity, leading to an increase in the EMC. The water molecules formed hydrogen bonds with the cellulose molecules, weakening the intermolecular forces between the cellulose fibers. This phenomenon manifested macroscopically as a reduction in the mechanical strength and stiffness of the sample [38,39].

#### 2.2.2. Desorption Process

**Figure 3:**
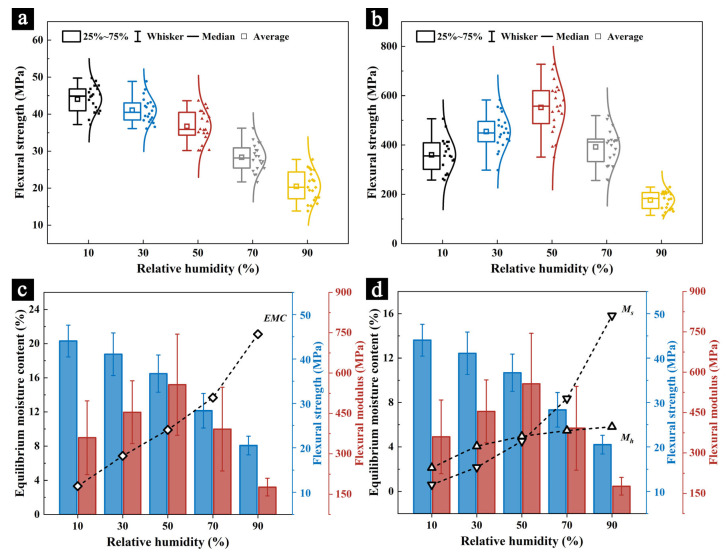
The flexural strength (**a**) and flexural modulus (**b**) of the samples at different humidity levels during the desorption process, along with the correlations between the flexural strength and modulus with the EMC (**c**), and $M_{h}$ and $\;M_{s}$ (**d**).

The TMA-MHG system was used to simulate and measure the changes in the mechanical properties of the samples as the humidity decreased, represented by the flexural strength (Figure 3a) and flexural modulus (Figure 3b). The results indicate that the TMA is a viable method for measuring the mechanical properties of precious and relatively fragile organic materials, such as palm leaf manuscripts. The trend in the changes to both the flexural strength and flexural modulus during the desorption process was consistent with the adsorption process: the flexural strength significantly decreased as the humidity increased and the flexural modulus decreased in response to both low and high humidity conditions.

However, different from this, the flexural strength and modulus during the desorption process were generally lower than during the adsorption process at the same relative humidity. For example, at 10% RH, the flexural strength and modulus during the adsorption process were 44.86 MPa and 380.38 MPa, respectively, while during the desorption process, they were 44.04 MPa and 360.06 MPa. At 90% RH, the flexural strength and modulus during adsorption were 22.03 MPa and 208.06 MPa, respectively, whereas during desorption, they dropped to 20.50 MPa and 176.83 MPa.

The isothermal moisture desorption curve of the samples (Figure 3c) and the variation of $M_{h}$ and $M_{s}$ with humidity (Figure 3d) further confirm the changes in the mechanical properties of the samples. Due to the hysteresis phenomenon, the EMC during the desorption process was consistently higher than during the adsorption process. The EMC values of the samples at 10% RH, 30% RH, 50% RH, 70% RH, and 90% RH during adsorption were 2.43%, 5.16%, 7.62%, 10.60%, and 18.85%, respectively, while during desorption, they were 3.33%, 6.85%, 9.85%, 13.67%, and 21.10%, respectively. This increase in moisture content led to a decrease in the mechanical strength and stiffness of the samples. Additionally, the results from the Hailwood–Horrobin (H–H) model suggest that this change is primarily driven by the increase in $M_{h}$, indicating that monolayer adsorption plays a significant role in the observed reduction in the mechanical properties.

#### 2.2.3. Hysteresis

**Figure 4:**
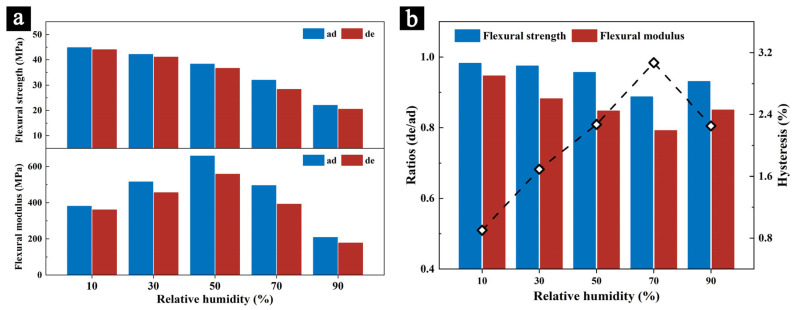
The changes in flexural strength and flexural modulus of the samples for different humidity levels during the two processes (**a**), and the correlation between these changes and the hysteresis of the samples (**b**).

A comparison of the flexural strength and flexural modulus of the samples subject to different relative humidity conditions during the two processes (Figure 4a) and their relationship with desorption hysteresis (Figure 4b) shows clear differences. As mentioned earlier, both the flexural strength and modulus during the desorption process are significantly lower than those during the adsorption process at the same relative humidity, which is caused by the hysteresis effect on the samples. The extent of the reduction in the flexural strength or modulus varies according to different humidity conditions.

The ratio of the flexural strength between the desorption and adsorption processes (de/ad) decreases as the humidity increases, with values of 0.98, 0.97, 0.95, 0.88, and 0.93, respectively, from low to high humidity. Similarly, the ratio of the flexural modulus (de/ad) follows the trend of 0.95, 0.88, 0.85, 0.79, and 0.85, showing a pattern of first decreasing and then increasing with increasing humidity. Both ratios reach their lowest point at 70% RH, indicating that the changes in the flexural strength and modulus are most pronounced at this humidity level.

Hysteresis typically represents the hygroscopic stability and adsorption efficiency of a material. The larger the hysteresis value, the slower the material absorbs or releases moisture when humidity fluctuates, and the more moisture it retains [36]. The ratio of the flexural strength and flexural modulus between the two processes is inversely proportional to the hysteresis value. In other words, the larger the hysteresis value at a particular humidity level, the greater the changes in the mechanical properties of the sample. This indicates that not only do the mechanical properties of the sample change with humidity fluctuations, but these changes are further amplified by the effect of hysteresis. If the sample is exposed to an environment with frequent humidity fluctuations over an extended period, continuous adsorption and desorption processes can lead to irreversible collapse of the pore structure in the samples, which, at the macroscopic level, results in reduced flexibility and decreased mechanical strength, thereby further impacting the overall mechanical performance of the material. Particularly in common indoor conditions ranging from 50% RH to 70% RH, palm leaf manuscripts may not only suffer from external mechanical damage, but could also experience internal stress due to changes in their mechanical properties. This internal stress may lead to issues, such as cracking and other forms of deterioration [28].

The results of the short-term study on the effects of relative humidity on the mechanical properties of the samples indicate that different humidity levels lead to changes in the EMC of palm leaf manuscripts, which in turn significantly affects their mechanical properties. Maintaining a stable environment in terms of humidity can prevent changes in the mechanical properties of palm leaf manuscripts, thereby preventing deterioration. Additionally, the samples generally exhibited better mechanical properties in relatively dry environments (≤50% RH), compared to higher humidity conditions (70% RH and 90% RH). This suggests that preserving palm leaf manuscripts in a drier environment would be a more ideal choice for their long-term conservation.

### 2.3. Long-Term Aging Effects of Relative Humidity on the Mechanical Properties of the Samples

#### 2.3.1. Aging Results of the Samples

**Figure 5:**
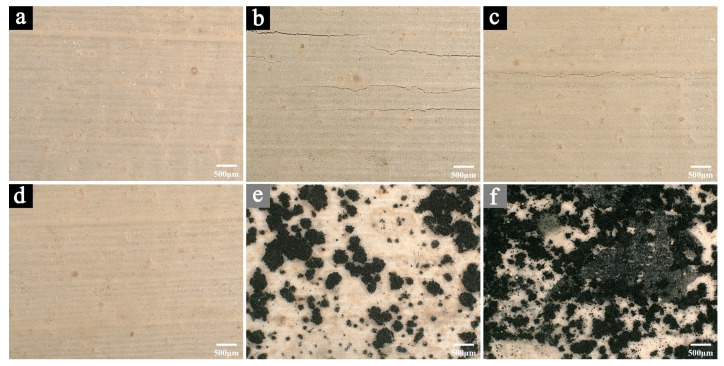
The microscopic images of the samples before aging (**a**) and after 100 days of aging in different humidity conditions: 10% RH (**b**), 30% RH (**c**), 50% RH (**d**), 70% RH (**e**), and 90% RH (**f**).

Figure 5 shows the microscopic morphological changes to the samples after 100 days of aging in different conditions (temperature of 25 °C and humidity levels of 10% RH, 30% RH, 50% RH, 70% RH, and 90% RH).

After 100 days of aging at 10% RH (Figure 5b) and 30% RH (Figure 5c), cracks appeared on the surface of the samples, with more severe cracking observed in the samples aged at 10% RH. In contrast, after 100 days of aging at 70% RH (Figure 5e) and 90% RH (Figure 5f), a significant amount of mold growth was observed on the surface of the samples, with the samples aged at 90% RH exhibiting more and denser mold colonies. Only the samples aged at 50% RH (Figure 5d) for 100 days showed no cracks or microbial damage, and their morphology did not significantly change compared to before aging.

#### 2.3.2. Mechanical Properties

**Figure 6:**
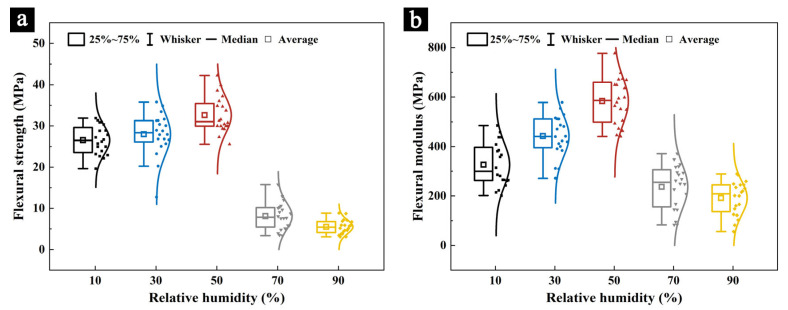
The flexural strength (**a**) and flexural modulus (**b**) of the samples after 100 days of aging in different humidity conditions.

The mechanical properties of the samples after 100 days of aging in different conditions were measured using the TMA, represented by the flexural strength (Figure 6a) and flexural modulus (Figure 6b). The results indicate that the TMA is a viable method for measuring the mechanical properties of precious and relatively fragile organic materials, such as palm leaf manuscripts.

After 100 days of aging, the flexural strength of the samples in different aging conditions (Figure 6a) decreased to different degrees. The flexural strength of the samples aged at 10% RH and 30% RH for 100 days was 26.46 MPa and 28.71 MPa, respectively, which may be due to the cracking phenomenon on the surface of the samples. After 100 days of aging at 70% RH and 90% RH, the flexural strength of the samples decreased significantly, reaching only 8.11 MPa and 5.51 MPa, respectively, which may be due to the degradation of the cellulose and hemicellulose components in the samples due to the growth of mold on the surface of the samples and their propagation activities [27]. The sample, after aging in 50% RH for 100 days, had the highest flexural strength (33.54 MPa) among all the aged samples, indicating that 50% RH had the least effect on the mechanical strength of the samples. After 100 days of aging, the change in the flexural modulus (Figure 6b) of the samples subject to different aging conditions was consistent with the change in the flexural strength and the sample, after 100 days of aging in 50% RH, had the highest flexural modulus (581.55 MPa) among all the aged samples.

#### 2.3.3. FT-IR

**Figure 7:**
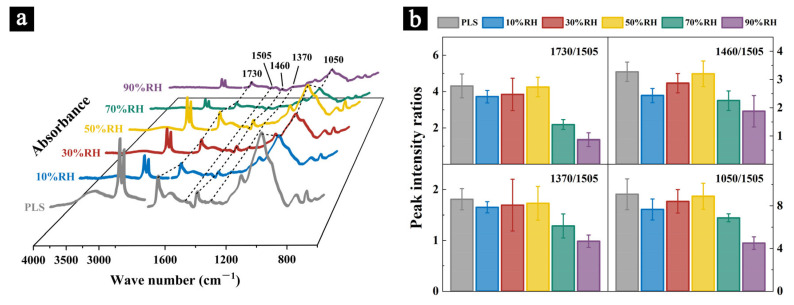
Infrared spectra of the samples before and after aging (**a**) and the ratio of characteristic peak intensities (**b**).

The infrared spectra of the samples after aging for 100 days subject to different aging conditions are shown in Figure 7a. The relative peak intensities obtained from the semi-quantitative analysis of the IR spectra of the samples are shown in Figure 7b.

The results indicate that after 100 days of aging in different conditions, the relative intensities of the characteristic peaks in the infrared spectra of the samples for cellulose and hemicellulose showed varying degrees of reduction. In the samples aged at 70% RH for 100 days, the peak intensity ratios I_1730_/I_1505_, I_1460_/I_1505_, I_1370_/I_1505_, and I_1050_/I_1505_ decreased by 49.32%, 31.21%, 29.04%, and 24.28%, respectively, compared to the values before aging. For samples aged at 90% RH for 100 days, these peak intensity ratios decreased by 68.62%, 42.62%, 45.61%, and 50.33%, respectively. This suggests that in high humidity environments, exacerbated by mold growth, the relative content of cellulose and hemicellulose in the samples decreases significantly, with greater degradation at higher humidity levels. Some studies have shown that the degradation of cellulose and hemicellulose can lead to a reduction in the mechanical properties of fibers, such as flexural strength and flexibility, potentially resulting in material damage related to physical performance [41]. Although the peak intensities of the samples aged at 10% RH and 30% RH for 100 days also decreased compared to the values before aging (with greater degradation observed in the 10% RH samples), the extent of the degradation was much less pronounced than in the high-humidity aged samples. This suggests that while dry environments do not significantly affect the relative content of cellulose and hemicellulose, prolonged exposure to excessively low humidity can cause irreversible issues, such as material shrinkage and surface cracking (as seen in Figure 5b,c), which also contribute to a decline in the material’s mechanical properties. After 100 days of aging at 50% RH, the peak intensity values of the samples decreased by only 1.39%, 2.14%, 4.42%, and 1.99%, respectively, compared to the values before aging, making these the least degraded among all the aged samples.

#### 2.3.4. XRD

**Figure 8:**
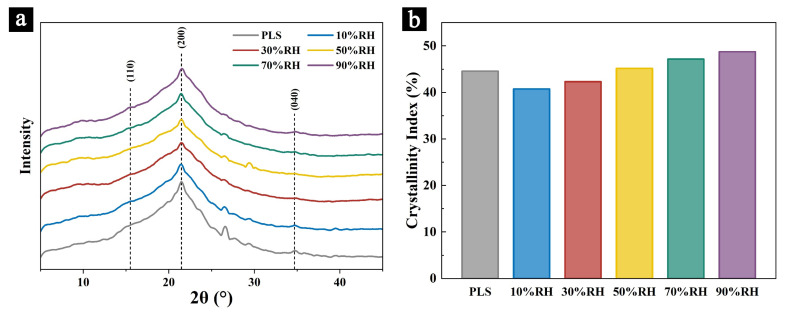
XRD patterns of the samples before and after aging (**a**) and the crystallinity index (**b**).

The XRD patterns and the calculated relative crystallinity results of the samples after aging for 100 days subject to different aging conditions are shown in Figure 8a and 8b, respectively.

The results show that the (200) main peak around 22° and the characteristic peaks at the (110) crystal plane (around 15°) and the (040) crystal plane (around 34°) in all the samples did not exhibit significant shifts in position. This indicates that the crystalline structure of cellulose in all the samples is Type I and aging in different relative humidity levels did not cause noticeable changes to the interplanar spacing or structural rearrangement. However, the (200) diffraction peaks of the samples aged in high humidity conditions (70% RH and 90% RH) were sharper, compared to those aged in low humidity conditions, and the amorphous regions appeared to be shorter. This suggests that the crystallinity of cellulose in the samples may have undergone varying degrees of change. Additionally, some of the observed non-cellulose diffraction peaks (e.g., at 26° and 29°) may originate from crystalline substances, such as Ca and Si, introduced during the preparation of the palm leaf manuscripts [7]. The calculated results on the relative crystallinity indicate that the crystallinity of the cellulose in the samples aged at 10% RH and 30% RH decreased compared to those before aging, likely due to minor cellulose degradation during the aging process. In contrast, the relative crystallinity of the cellulose in the samples aged at 70% RH and 90% RH increased compared to the pre-aging state. This suggests that while high humidity and mold invasion lead to significant degradation of both cellulose and hemicellulose, the amorphous regions, such as hemicellulose, degrade at a faster rate and to a greater extent, resulting in an apparent increase in the relative crystallinity of cellulose. This observation is consistent with the phenomena observed in the infrared spectroscopy analysis of the samples. In comparison, the crystallinity of the cellulose in the samples aged at 50% RH showed minimal changes, indicating a more stable structure.

The results of the study on the long-term aging effects of relative humidity on the mechanical properties of the samples show that excessively dry environments lead to surface cracking in palm leaf manuscripts, while humid environments result in mold growth and the degradation of key chemical components. Although the causes differ, both environments ultimately result in significant changes to the mechanical properties of palm leaf manuscripts, indicating that neither environment is suitable for their preservation. In contrast, the samples aged at 50% RH exhibited no visible damage, and their mechanical properties and chemical structure remained largely unchanged. This suggests that 50% RH is a relatively optimal humidity condition for the preservation of palm leaf manuscripts.

## 3. Materials and Methods

### 3.1. Experimental Materials

**Figure 9:**
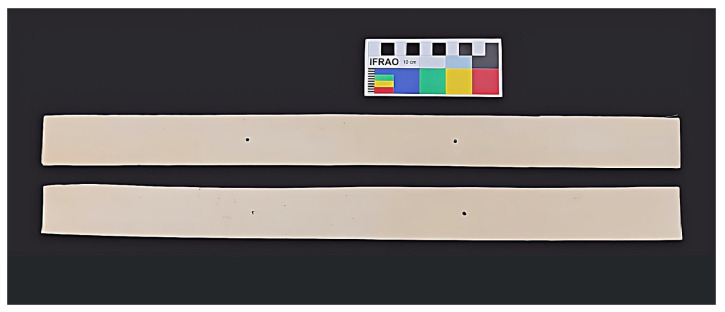
Photograph of a raw palm leaf manuscript sample (PLS).

The experimental samples in this study were raw palm leaf manuscripts from Yunnan Province, China. These samples were produced following the traditional palm-leaf manuscript-making process, which has been recognized as part of China’s national intangible cultural heritage. The preparation involved boiling, washing, air drying, trimming, and flattening the leaves from the talipot palm (*Corypha umbraculifera* L.) tree [11], resulting in the creation of the samples used in this study. This research focuses on the impact of relative humidity on the mechanical properties of the palm leaves themselves, the core material of palm leaf manuscripts. Therefore, the raw palm leaf manuscript samples (PLSs) were not subjected to subsequent treatments, such as writing or coloring, in order to avoid any influence from pigments or binding materials on the study results. A photograph of a raw palm leaf manuscript sample is shown in Figure 9.

### 3.2. Simultaneous DVS

The equilibrium moisture content (EMC) of the samples, as well as the isothermal moisture adsorption and desorption curves within the tested humidity range, were measured using a high-throughput dynamic vapor sorption analyzer (SPSx-1μ, ProUmid, Ulm, Germany) subject to the same temperature, but different relative humidity conditions. The testing range was 0% to 95% RH, with humidity increments of 10% RH between 0% and 90% RH, and 5% RH between 90% and 95% RH. The temperature was maintained at 25 °C. The equilibrium conditions were defined as a weight change of less than 0.1% within 10 min, or a maximum measurement time of 360 min, per humidity gradient.

### 3.3. Sorption Models

The hygroscopic test results were fitted using the classic Hailwood–Horrobin (H–H) model and the model parameters were calculated using software (SPSS Statistics 22, IBM, Armonk, NY, USA) to further explain the hygroscopic characteristics of the samples.

The H–H model equation is as follows:

(1) $$EMC=M_{h}+M_{s}=\frac{1800}{w}\cdot \frac{k_{1}\cdot k_{2}\cdot RH}{100+k_{1}\cdot k_{2}\cdot RH}\cdot 100\%+\frac{1800}{w}\cdot \frac{k_{2}\cdot RH}{100-k_{2}\cdot RH}$$

where EMC (g/g) is the equilibrium moisture content; RH (%) is the relative humidity; $M_{h}$ is the monolayer moisture content (%); $M_{s}$ is the multilayer moisture content (%); w is the molecular weight of the materials at each adsorption site; and $k_{1}$ and $k_{2}$ are equilibrium constants in the sorption process [31].

### 3.4. Flexural Strength Test

**Figure 10:**
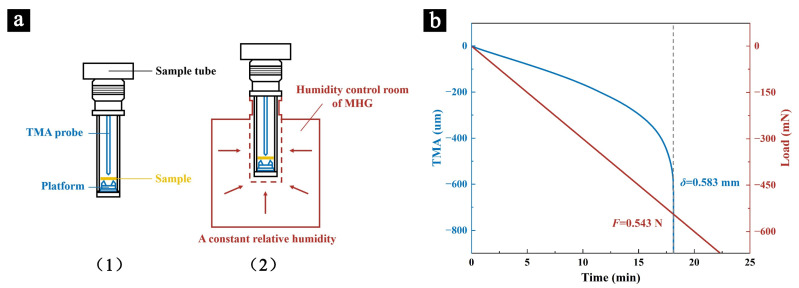
A schematic diagram of the TMA’s flexural strength test components (**a1**) and the humidity application process (**a2**), along with the load/displacement–time curves during the testing process (**b**).

The flexural strength of the samples was tested using a thermomechanical analyzer (TMA 7100, Hitachi, Chiyoda, Japan), equipped with quartz probes and components, as shown in Figure 10(a1). To investigate the short-term effects of relative humidity on the mechanical properties of palm leaf manuscripts, a custom-made humidity control chamber was connected to the TMA. The stable relative humidity environment was provided by a Modular Humidity Generator (MHG 32, ProUmid, Ulm, Germany), as illustrated in Figure 10(a2).

Two relative humidity control programs were used to simulate the changes in the samples during the adsorption and desorption phases, as follows:

- Adsorption phase: After controlling the relative humidity at 0% RH for 6 h, the humidity was increased to the set levels (10% RH, 30% RH, 50% RH, 70% RH, and 90% RH) and held for 6 h;
- Desorption phase: After controlling the relative humidity at 95% RH for 6 h, the humidity was decreased to the set levels (same as above) and held for 6 h.

After completing these phases, the flexural strength test was conducted at a constant temperature of 25 °C, with the tested humidity set to the corresponding levels. The initial load was 0.1 mN and the loading rate was 30 mN/min, continuing until the sample fractured and the test curves were obtained (Figure 2b). The samples were cut to approximately 8 mm × 1 mm, with 20 samples tested under each humidity condition.

For the aged samples, no humidity was applied during testing. The TMA was used to test the samples directly. Similarly, the samples were cut to approximately 8 mm × 1 mm, with 20 samples tested under each aging condition.

The flexural strength (σ) and flexural modulus (E) of the samples were calculated using Formulas (2) and (3), respectively:

(2) $$\sigma =\frac{3FL}{2bd^{2}}$$

(3) $$E=\frac{FL^{3}}{4\delta bd^{3}}$$

where  σ  (MPa) and E (MPa) are the flexural strength and flexural modulus of the sample, respectively; *F* (N) is the maximum load at the point of sample fracture; *L* (mm) is the span between the supports on the TMA’s flexural strength testing platform, fixed at 5 mm; *b* (mm) and *d* (mm) are the width and thickness of the sample, respectively, measured using a super-depth microscope (VHX-6000, KEYENCE, Osaka, Japan); δ (mm) is the maximum displacement of the sample at the point of fracture.

### 3.5. Simulated Aging Experiment

A sufficient number of raw palm leaf manuscript samples (PLSs) were divided into five groups, each containing 30 individual samples. The five groups of samples were placed in an environmental test chamber (GSH-64, Espec, Osaka, Japan), where different aging conditions were set (all at a temperature of 25 °C, with humidity levels of 10% RH, 30% RH, 50% RH, 70% RH, and 90% RH). After 100 days of aging, the samples were removed and their morphological changes were observed using a super-depth microscope (VHX-6000, KEYENCE, Osaka, Japan). Some of the samples were then selected for flexural strength testing and chemical structure analysis.

### 3.6. FT-IR Test

The aged samples were finely ground and passed through a 200-mesh sieve. They were then mixed with KBr (spectrally pure, Macklin, Shanghai, China) at a mass ratio of 1:100 and pressed into pellets. The chemical structure of the samples was characterized using Fourier transform infrared spectroscopy (Nicolet™ iS™5, Thermo Scientific, Waltham, MA, USA), to compare the differences in the characteristic functional groups before and after aging. The absorption spectra were recorded in the range of 4000–800 cm^−1^, with baseline corrections performed at 4000, 1890, 1530, and 864 cm^−1^ [42]. Three samples were tested for each aging condition.

In order to further characterize the changes to the chemical structure of the samples, the infrared spectra of the samples were analyzed semi-quantitatively, using the peak intensity method. The absorption peaks at 1730 cm^−1^ (stretching vibration by the carbonyl group on hemicellulose) and 1460 cm^−1^ (stretching vibration by the methylene group on hemicellulose) were selected as the characteristic peaks of hemicellulose, and the absorption peak at 1505 cm^−1^ (backbone vibration of butyl propane in butyl lignin) was chosen to represent lignin, and the absorption peaks at 1370 cm^−1^ (bending vibration by the methyl group on cellulose) and 1050 cm^−1^ (bending vibration by the glycosidic bond on cellulose) were chosen to represent cellulose [43,44,45]. The intensity values of the infrared spectral peaks were measured after baseline correction using software (OMNIC 9.2, Thermo Scientific, USA).

### 3.7. XRD Test

The crystal structure and crystallinity of the aged samples were analyzed using an X-ray diffractometer (D8 ADVANCE, Bruker, Munich, Germany). The operational settings included a working voltage of 40 kV, a current of 40 mA, and the use of Cu-Kα radiation (wavelength λ = 1.5406 Å), as the radiation source. The scanning angle range during measurement was set from 5° to 50° (2θ), with a scanning speed of 1°/min and a step size of 0.02°. The diffraction patterns were fitted using software (MDI Jade 9, ICDD, Newtown Square, PA, USA) and the crystallinity index (*CI*) of the samples was calculated using the Segal method.

(4) $$CI=\frac{I_{200}-I_{am}}{I_{200}}\times 100\%$$

where *CI* is the crystallinity index of the sample; $I_{200}$ is the maximum intensity of the lattice diffraction angle of (200) near 2θ = 22.4°, which signifies both the crystalline and non-crystalline regions; and $I_{am}$ is the minimum intensity near the 2θ angle of 18°, indicating the non-crystalline region.

## 4. Conclusions

This study shows that varying levels of relative humidity affect the EMC and significantly impact the mechanical properties of palm leaf manuscripts. The flexural strength decreased notably with higher humidity, while the flexural modulus initially increased and then decreased as the humidity rose. Hysteresis caused a reduction in both the flexural strength and modulus during desorption compared to adsorption, with larger hysteresis values resulting in greater changes to the mechanical properties. Therefore, maintaining a stable environment in regard to humidity can help preserve the mechanical integrity of palm leaf manuscripts and prevent deterioration.

Long-term exposure to either very dry or humid conditions harms the mechanical properties of palm leaf manuscripts, making both conditions unsuitable for preservation. Dry conditions lead to surface cracking, while humid conditions promote mold growth, which degrades the manuscripts’ primary chemical components. Samples aged at 50% RH showed no visible damage, with the material’s mechanical properties and chemical structure largely preserved, suggesting that 50% RH is an optimal humidity condition for preserving palm leaf manuscripts.

This study used DVS and TMA-MHG methods to examine changes in the mechanical properties of palm leaf manuscripts subject to various humidity conditions, assessing both the short-term effects and long-term aging. The results provide a comprehensive and in-depth understanding of the characteristics of palm leaf manuscripts, aiding in the development of more effective preventive conservation strategies. It is important to note, however, that the preservation of palm leaf manuscripts is often influenced by a combination of temperature and humidity. The recommendation of 50% RH as a suitable humidity condition is based on preliminary data from this study. Future research should explore the combined effects of more complex environmental factors on both the mechanical and other properties of palm leaf manuscripts. In summary, this study provides valuable data that support the long-term preservation and preventive conservation of these priceless manuscripts, providing a useful reference for further research.

## Figures and Tables

**Table 1:** The H–H model parameters for the samples.

Process	*w*	$k_{1}$	$k_{2}$	R^2^
ad	415.087	14.717	0.869	0.999
de	297.972	9.740	0.746	0.997

In the table, ad represents the adsorption process; de represents the desorption process; *w* is the molecular weight of the wood at each adsorption site; and $k_{1}$ and $k_{2}$ are the equilibrium constants related to monolayer and multilayer adsorption, respectively.

## Data Availability

The data presented in this study are available on request from the corresponding author.
